# Association between differential somatic cell count and California Mastitis Test results in Holstein cattle

**DOI:** 10.3168/jdsc.2022-0249

**Published:** 2022-09-30

**Authors:** Che-Hsuan Huang, Nobuyuki Kusaba

**Affiliations:** Field Center of Animal Science and Agriculture, Obihiro University of Agriculture and Veterinary Medicine, Obihiro, Hokkaido, Japan 080-8555

## Abstract

The California Mastitis Test (CMT) has been used to estimate total somatic cell count (SCC) levels in milk; however, milk with similar SCC levels occasionally shows inconsistent CMT results, which limits the use of the CMT. This observational study aimed to investigate how differential cell counts in milk influence the CMT in Holstein cattle through the novel parameters differential somatic cell count (DSCC) and macrophage proportion (MAC). We performed the CMT on d 0, 3, 5, 7, 14, and 21 after identifying mastitis, and simultaneously measured SCC, DSCC, and MAC at the quarter level. We followed 58 mastitis events occurring in 41 cows and obtained 307 quarter-level records after data cleaning. We transformed SCC to somatic cell score (SCS) and MAC to its logarithm to fit the normal distribution and analyzed the data using the cumulative logit mixed model. Results showed that both an increase in SCS (odds ratio: 3.66, 95% confidence interval: 2.89–4.64) and the logarithm of MAC (odds ratio: 4.35, 95% confidence interval: 1.91–9.91) can contribute to a higher CMT score. During the healing process of mastitis, MAC tends to increase as SCC decreases; thus, even samples with low SCC can cause positive CMT reactions. We recommend that practitioners avoid making treatment decisions based on the CMT alone. We also noted that the CMT is sensitive to subclinical mastitis with high MAC, hence it could be considered an alternative to detecting high MAC (chronic) mastitis.

## Highlights

• Differential cell counts in milk affect the California Mastitis Test (CMT) result.
• Changes in macrophage proportions cause discrepancies between CMT scores and SCC.
• Using the CMT alone for monitoring clinical outcomes of mastitis may be unsuitable.
• Variation in differential cell counts between cases limits the use of the CMT.

## Graphical Abstract

**Figure:**
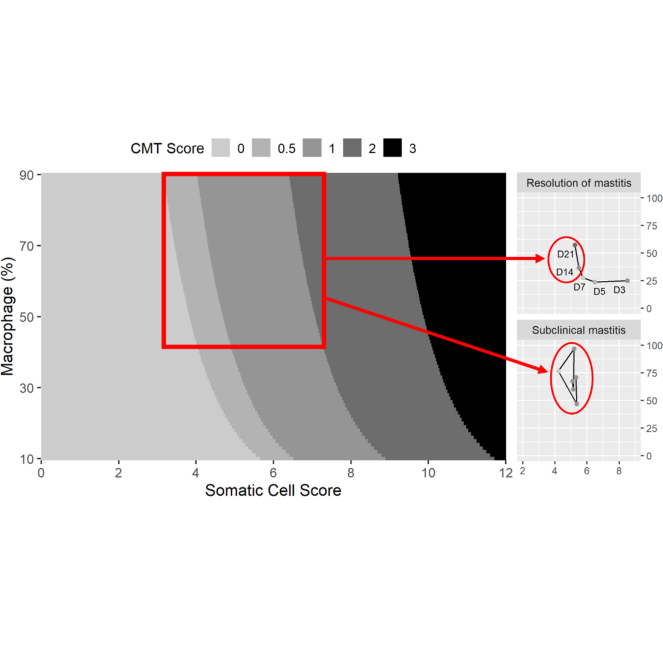
**Summary:** The left panel shows the estimated California Mastitis Test (CMT) scores as a function of somatic cell score (SCS) and macrophage proportions. Because the SCS is a logarithmic conversion of somatic cell counts (SCC), the same CMT reaction may represent hugely varied SCC values depending on the macrophage proportions. As shown in the 2 right panels, elevated macrophage proportions have been identified in the resolution of mastitis and some subclinical mastitis cases. In such situations, the interpretation of the CMT scores requires more consideration.

A reliable test rapidly identifying quarters with subclinical mastitis is warranted for mastitis control. The California Mastitis Test (**CMT**) is a fast and cheap cow-side test (Schalm, 1957) widely used for screening IMI (Sargeant et al., 2001), guiding selective dry cow therapy (**SDCT**; Bhutto et al., 2012), and monitoring therapeutic response in clinical mastitis (Lam et al., 2009). When the CMT reagent, a detergent, mixes with milk samples with cellular contents, it lyses cell membranes, precipitating cell DNA and proteins and resulting in increased viscosity (Nageswararao and Derbyshire, 1969). This reaction can be scored on an ordered categorical scale, allowing cell numbers (i.e., SCC) in milk to be estimated. As an elevation of SCC (>200,000 cells/mL) is a strong indication of IMI (Dohoo and Leslie, 1991), treatment decisions have been facilitated through the CMT. Nonetheless, the CMT reaction is not a precise indicator of SCC (Ruegg and Reinemann, 2002) and has lower accuracy for detecting IMI compared with SCC tests (Sargeant et al., 2001). Consequently, in clinical practice, using the CMT alone may result in improper treatment decisions.

Because the CMT works through the precipitation of cellular DNA and proteins, and structural differences exist between cell types, proportions of each cell type in milk likely influence the CMT. The immune cells in milk, including PMN, macrophages, and lymphocytes (Sordillo et al., 1997), play different roles in immune responses, and their proportions vary between stages of mastitis (Leitner et al., 2000). As a result, CMT results may vary depending on the stage of mastitis. Recently, a new method has been developed to efficiently determine differential cell counts in milk (Damm et al., 2017), which measures the combined proportions of PMN and lymphocytes, namely differential SCC (**DSCC**); macrophage proportions (**MAC**) can be calculated by the formula 100 − DSCC. Studies have reported that DSCC also varies between stages of mastitis (Kirkeby et al., 2021).

This observational study aimed to investigate how differential cell counts in milk affect the CMT in Holstein cattle through the novel parameters DSCC and MAC. We performed the CMT on 0, 3, 5, 7, 14, and 21 d after identifying mastitis, and simultaneously collected milk samples from the affected quarter to determine SCC, DSCC, and MAC. With this approach, we can understand how varied total and differential cell counts between stages of mastitis affect the CMT result. We hypothesized that both total and differential cell counts influence the CMT. The conclusion may be useful for understanding the variation in the CMT results between stages of mastitis.

All procedures performed were approved by the Animal Care and Use Committee of Obihiro University of Agriculture and Veterinary Medicine (**OUAVM**; Permission number 21–156). We followed the statement from Strengthening the Reporting of Observational Studies in Epidemiology-Veterinary (STROBE-Vet; Sargeant et al., 2016) to report this study. The study used unpublished data originally collected for evaluating the effect of oral supplementation of chitosan on mastitis. The methodology of the original study was not considered to affect the objective of the current study. The study was conducted at the Field Center of Animal Science and Agriculture at OUAVM from June 2021 to February 2022. The study herd consisted of 70 Holstein cows, kept in a freestall barn, fed a TMR, milked twice daily in a parlor, and with an average annual milk production of 11,000 kg. Bulk tank SCC of the herd ranged from 100,000 to 300,000 cells/mL throughout the study period. Cows confirmed with clinical or subclinical mastitis were considered for enrollment, but cows with mastitis caused by the same pathogen as the previous occurrence (i.e., recurrent mastitis) were later excluded. Clinical mastitis was first identified by farm staff during milking, through signs such as abnormal milk or swollen udder, and confirmed by study personnel within 6 h. Subclinical mastitis was first identified by the monthly DHI test (>200,000 cells/mL in composite milk), and the affected quarter was further identified through the CMT. Cows with clinical mastitis caused by major pathogens (i.e., *Staphylococcus aureus*, streptococci and related genera, gram-negative rods) were treated with antibiotics once a day following afternoon milking for 5 consecutive days (i.e., from d 1 to 5), but other cows remained untreated.

Concurrent with the confirmation of mastitis, the quarter milk sample was collected (d 0) and underwent the CMT after cleaning the udder and forestripping. A 30- to 50-mL sample was preserved at 4°C and sent to an accredited laboratory within 4 d to determine SCC (cells/mL), DSCC (%), and MAC (%) using the Fossomatic 7 DC (Foss Analytical A/S). Another 3 to 5 mL of sample was collected aseptically to determine infection status through bacterial culture. Bacterial culture was performed immediately after collection, following National Mastitis Council guidelines (NMC, 2017) with some modifications, in which 50 µL of milk was cultured instead of the usual 10 µL. Bacteria were categorized through morphology, hemolysis patterns, Gram staining, and biochemical tests (e.g., catalase, coagulase, indole, and oxidase tests). On 3, 5, 7, 14, and 21 d after identifying mastitis, the same procedure (i.e., the CMT, bacterial culture, and sampling for SCC, DSCC) was conducted shortly before afternoon milking.

The CMT was performed with a detergent (8.5 g/L sodium dodecylbenzene sulfonate) containing bromothymol blue (P.L. tester; Nippon Zenyaku Kogyo Co. Ltd.), following a standard method (Schalm, 1957). Milk was injected into a paddle and adjusted to 3 mL. An equal volume of CMT reagent was added and mixed well. The reaction was interpreted within 15 s and scored from 0 to 3, where 0 was no thickening, 0.5 (trace reaction) was slight thickening, 1 was thickening without gel formation, 2 was immediate thickening with slight gel formation, and 3 was distinct gel formation. All CMTs were performed by the same trained individual, who was blinded to SCC and DSCC values when conducting the CMT.

All statistical analyses were performed using R software (v4.0.5; R Core Team, 2021). We excluded mastitis events from the analysis where follow-up was interrupted due to culling (n = 1), dry-off (n = 2), or receiving systemic medications for lameness (n = 2). Somatic cell count was unmeasurable in milk full of clots (n = 4), and DSCC measurements were unreliable in samples with ≤50,000 cells/mL (n = 37); thus, such records were also excluded. The SCC values were transformed to SCS to fit a normal distribution. Visual inspection of the quantile-quantile plots for DSCC and MAC values indicated left-skewed and right-skewed distributions, respectively. Because MAC is equivalent to 100 – DSCC, MAC was log-transformed and used for analysis (Osborne, 2002). To determine the association between the SCS, MAC, and CMT score (an ordinal categorical outcome), we analyzed data in a cumulative logit mixed model with proportional-odds assumption using the “clmm” function within the “ordinal” package (Christensen, 2019). The model was of the form

Logit [*P*(*Y_i_* ≤ *j*)] = *θ_j_* – β_1_*SCS_i_* – β_2_log(*MAC_i_*) – *day_k_* – *γ_m_* − *ε_i_*,

where *Y_i_* is the response variable (CMT score) for the *i*th observation, which may take on a value of 0, 0.5, 1, 2, or 3, and *j* denotes the 5 possible values of *Y_i_*. The parameter *θ_j_* provides a separate intercept for each category *j*; *SCS_i_, MAC_i_*, and *day_k_* are fixed effects of SCS, MAC, and sampling day (6 levels: d 0, 3, 5, 7, 14, 21), respectively, for the *i*th observation on day *k*. β_1_ and β_2_ are regression coefficients for *SCS_i_* and *MAC_i_*, respectively; *γ_m_* is the random effect allowing random intercept for each mastitis event to account for the correlation of effects within mastitis event; and *ε_i_* is the error term. The significance of each term in the model was tested by likelihood ratio tests (*P* ≤ 0.05). The random effect of each cow and 2-way interactions of the fixed effects were also tested but later dropped from the model due to insignificance.

We followed 58 mastitis events occurring in 41 cows and obtained 348 quarter-level records. After excluding data where SCC or DSCC values were inaccessible, 307 records were included in the final data set. Descriptive statistics of SCS and DSCC (%) by CMT scores are given in Table 1. Despite a positive association between SCS and CMT scores, SCS varied greatly in each score, indicating that the scores may not depend solely on SCS. All missing values in category CMT score 0 were due to the unavailability of DSCC values and with SCS ≤2; thus, the mean of SCS here was overestimated. However, these missing values were not considered to affect the estimation of the model, because samples with SCS ≤2 are assumed to show negative reactions in the CMT regardless of differential cell counts.

**Table 1:** Descriptive statistics of SCS and differential SCC (DSCC) by each score of California Mastitis Test (CMT; n = 307)

CMT score	n	NA^1^	SCS			DSCC		
			Mean	SD	Minimum	Maximum	Mean, %	SD
0	102	36	4.48	1.45	2.16	8.77	69.74	13.3
0.5	58	1	5.97	1.56	2.64	10.00	71.58	10.0
1	78	0	7.07	1.51	4.00	11.09	72.25	14.5
2	45	1	8.85	1.57	5.00	11.67	71.28	17.1
3	24	3	11.25	0.92	9.56	12.37	69.59	7.3

1 NA = missing values (n = 41).

The model summary and ANOVA results are shown in Table 2. Both SCS and MAC significantly influenced CMT results (*P* = 2.71E-44 and *P* = 5.08E-4 in ANOVA, respectively). Specifically, CMT scores were positively associated with the logarithm of MAC (odds ratio: 4.35, 95% CI: 1.91–9.91). This phenomenon can be explained by different cellular structures between macrophages and PMN. Nagahata et al. (1987) reported that the activity of lysosomal enzymes (i.e., *N*-acetyl-β-d-glucosaminidase and β-glucuronidase) is higher in milk macrophages than in milk PMN, and the activity of these enzymes was strongly related to the CMT reaction. The difference in the activity of lysosomal enzymes between macrophages and PMN could be a potential mechanism causing their distinct behavior in the CMT.

**Table 2:** Summary of the cumulative logit mixed model, including estimated coefficients (SE) and odds ratio (95% CI), and results from ANOVA, including likelihood ratio χ^2^ value (LR χ^2^), df, and *P*-value

Parameter	Coefficient (SE)	Odds ratio (95% CI)	ANOVA		
			LR χ^2^	df	*P*-value
SCS	1.30 (0.12)	3.66 (2.89, 4.64)	194.90	1	2.71E-44
log (MAC)^1^	1.47 (0.42)	4.35 (1.91, 9.91)	12.09	1	5.08E-4
Days after identifying mastitis			11.10	5	0.049
0	Referent				
3	−1.58 (0.51)	0.21 (0.08, 0.56)			
5	−1.12 (0.53)	0.33 (0.12, 0.92)			
7	−1.48 (0.56)	0.23 (0.08, 0.69)			
14	−1.39 (0.58)	0.25 (0.08, 0.78)			
21	−1.11 (0.58)	0.33 (0.11, 1.02)			
Intercepts of cut-points					
0 | 0.5	10.31 (1.95)				
0.5 | 1	12.11 (1.99)				
1 | 2	14.99 (2.07)				
2 | 3	18.62 (2.23)				

1 Logarithm of macrophage proportions.

Compared with samples collected on the day mastitis was identified (i.e., d 0), samples collected on other days tended to have a lower CMT score. This can be attributed to a different sampling timing (d 0 samples were collected after milking) or expectation from the investigator (expecting that quarters with mastitis would have high CMT scores). Finally, the random effect of mastitis events was highly significant (*P* = 1.49E-4), suggesting that variance in CMT results exists between mastitis events.

To explain the significance of these findings, we selected 4 cases to illustrate how changes in SCS and macrophage proportions affect CMT results (Figure 1). In cases 912B and 922B (upper 2 panels), SCS decreased gradually with a corresponding increase in MAC, indicating a resolution of mastitis (Sládek and Rysanek, 2001). This was supported by the negative bacterial culture results on d 7, 14, and 21 in both cases. However, in case 922B, the CMT result would lead us to the opposite conclusion. Specifically, the CMT showed positive reactions on d 14 and 21, but SCS had been decreasing gradually since d 7 in this case. This conflicting finding is thought to arise from the increasing macrophage proportions during the healing process of mastitis, leading to high CMT scores at relatively low levels of SCC. Because the CMT result conflicted with bacterial culture results and SCC, the treatment period would have been extended unnecessarily if the treatment decision were based solely on CMT.

**Figure 1:**
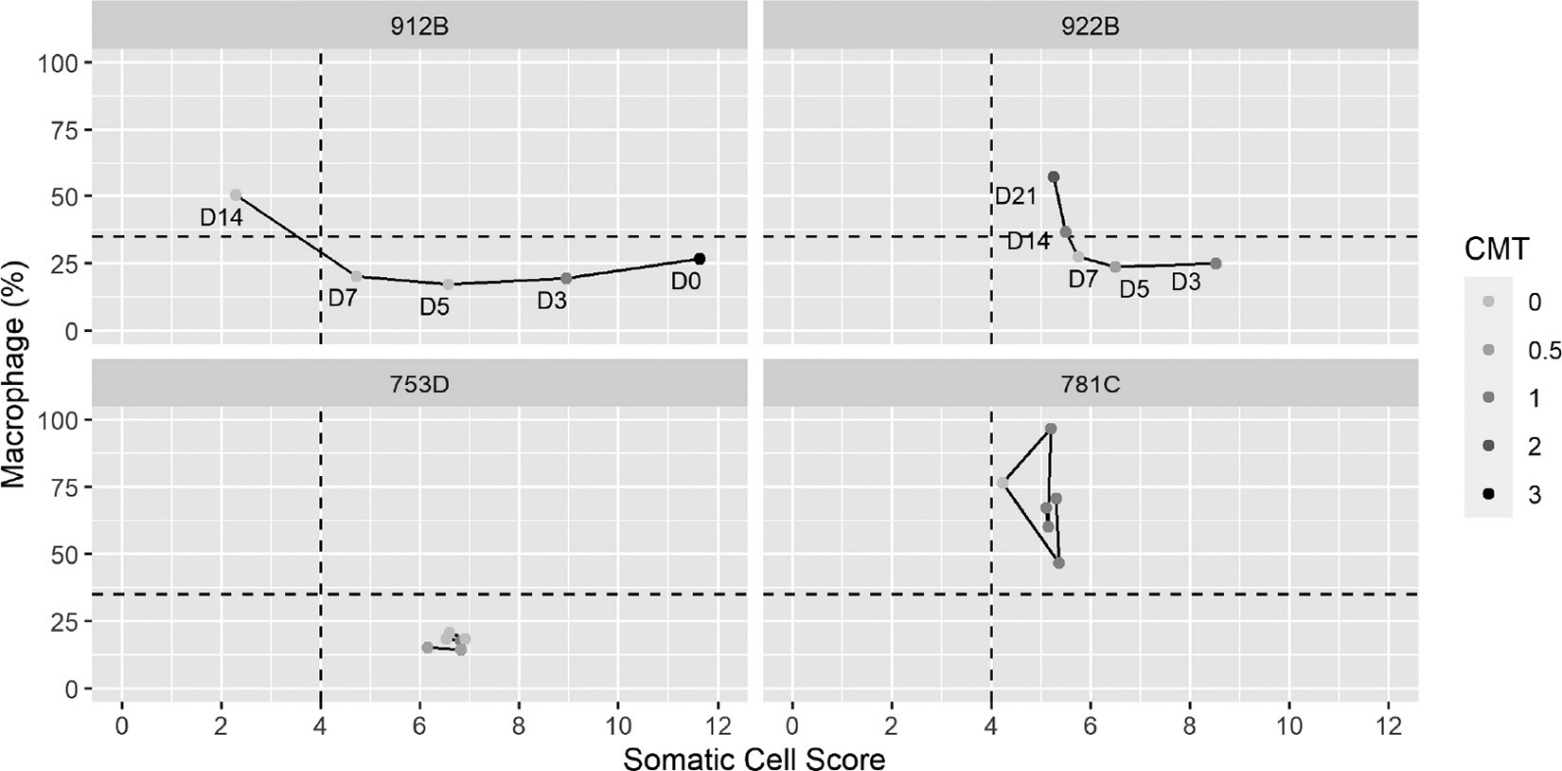
Examples of how changes in SCS and macrophage proportions affect California Mastitis Test (CMT) results in 4 different cases (912B, 922B, 753D, and 781C). The 2 reference lines correspond to where SCS equals 4 and macrophage proportions equal to 35% (i.e., differential SCC equal to 65%). In the upper 2 panels, points are labeled with days (D) after identifying mastitis.

The disagreement between CMT result and SCC level existed not only in mastitis resolution. Cases 753D and 781C (lower 2 panels in Figure 1) were cases of subclinical mastitis untreated with antibiotics. An IMI caused by *Streptococcus* spp. had been detected persistently in 753D, whereas no IMI had been detected in 781C (i.e., nonspecific subclinical mastitis) during the study period. Although case 753D had continually higher SCS than case 781C, the latter showed stronger reactions in the CMT. This discrepancy probably resulted from the distinct differential cell counts between the 2 cases. Studies have reported DSCC values to be higher in cows with IMI caused by major pathogens compared with those in cows without IMI or with IMI caused by minor pathogens (Schwarz et al., 2020b; Kirkeby et al., 2021), consistent with the current observation. If the CMT was used for guiding an SDCT program, for instance, antibiotics may be allocated to quarters without IMI. This could lead to higher antimicrobial use in a CMT-guided SDCT than in an SCC-guided SDCT (McDougall et al., 2022).

Additionally, the 2 dotted reference lines in Figure 1 were drawn based on cut-offs suggested by Schwarz et al. (2020a), in which they categorized cows into the following 4 udder health groups: healthy = SCS ≤4 and DSCC ≤65%; suspicious mastitis = SCS ≤4 and DSCC >65%; mastitis = SCS >4 and DSCC >65%; and chronic mastitis = SCS >4 and DSCC ≤65%. Case 781C would be grouped into “chronic mastitis” according to this definition. Those authors reported that this group of cows are the least productive and the most likely to leave the herd (Schwarz et al., 2020a, 2021); thus, identifying this group of cows and quarters potentially benefits herd management, and CMT can be a screening tool when quarter-level information is inaccessible.

There are some limitations in this study. First, because we used the CMT to identify quarters with subclinical mastitis, selection bias may have been introduced. Another potential source of bias is the expectation of the CMT reaction based on mastitis progression; thus, we considered sampling days from identifying mastitis to estimate this effect. Potential confounders can be milk components correlated with DSCC (e.g., lactose; Bobbo et al., 2020; Pegolo et al., 2021); however, milk components other than nucleated cells are less likely to affect the CMT reaction, in which polymerized DNA is required (Nageswararao and Derbyshire, 1969). Notably, our findings require further validation by performing the CMT with other reagents, because the choice of reagent is known to affect the sensitivity of the CMT (Leach et al., 2008). We selected several cases to illustrate how changes in macrophage proportions can affect the field use of the CMT, but the generalizability of these findings warrants further research.

In conclusion, our results indicate a positive association between the proportion of macrophages in SCC and the CMT result. In the recovery of mastitis, MAC tended to increase as SCC decreased, possibly leading to a false-positive CMT reaction and, consequently, an unnecessary extension of treatment. Thus, we advise not making treatment decisions based on the CMT alone. Instead, bacterial culture and SCC or DSCC tests may be helpful aids. On the other hand, we noted that the CMT is especially sensitive to quarters with chronic mastitis. Therefore, the CMT can be considered a screening tool for identifying cows with chronic mastitis.
